# Genome-Wide Identification and Expression Analysis of the Tubby-Like Protein Family in the *Malus domestica* Genome

**DOI:** 10.3389/fpls.2016.01693

**Published:** 2016-11-14

**Authors:** Jia-Ning Xu, Shan-Shan Xing, Zheng-Rong Zhang, Xue-Sen Chen, Xiao-Yun Wang

**Affiliations:** State Key Laboratory of Crop Biology, College of Life Science, Shandong Agricultural UniversityTaian, China

**Keywords:** tubby-like protein, bioinformatics, signature motifs, abiotic stress, apple

## Abstract

Tubby-like proteins (TLPs), which have a highly conserved β barrel tubby domain, have been found to be associated with some animal-specific characteristics. In the plant kingdom, more than 10 TLP family members were identified in *Arabidopsis*, rice and maize, and they were found to be involved in responses to stress. The publication of the apple genome makes it feasible to systematically study the TLP family in apple. In this investigation, nine TLP encoding genes (TLPs for short) were identified. When combined with the TLPs from other plant species, the TLPs were divided into three groups (group A, B, and C). Most plant TLP members in group A contained an additional F-box domain at the N-terminus. However, no common domain was identified other than tubby domain either in group B or in group C. An analysis of the tubby domains of *Md*TLPs identified three types of conserved motifs. Motif 1 and 2, the signature motifs in the confirmed TLPs, were always present in *Md*TLPs, while motif 3 was absent from group B. Homology modeling indicated that the tubby domain of most *Md*TLPs had a closed β barrel, as in animal tubby domains. Expression profiling revealed that the *Md*TLP genes were expressed in multiple organs and were abundant in roots, stems, and leaves but low in flowers. An analysis of *cis*-acting elements showed that elements related to the stress response were prevalent in the promoter sequences of *MdTLP*s. Expression profiling by qRT-PCR indicated that almost all *MdTLP*s were up-regulated at some extent under abiotic stress, exogenous ABA and H_2_O_2_ treatments in leaves and roots, though different *Md*TLP members exhibited differently in leaves and roots. The results and information above may provide a basis for further investigation of TLP function in plants.

## Introduction

Tubby-like proteins (TLPs) are present in all eukaryotes, from single-celled to multicellular organisms (Liu, 2008), including *Caenorthabditis elegans, Drosophila, Arabidopsis*, rice, maize, chicken, and mouse (North et al., 1997; Heikenwalder et al., 2001; Ronshaugen et al., 2002; Figlewicz et al., 2004). TLPs have a typical tubby domain that forms a closed β barrel with 12 anti-parallel strands and a central hydrophobic α helix (Boggon et al., 1999). The number of TLPs ranges from 4 in humans to 15 in maize (Stone and Callis, 2007; Yulong et al., 2016). The distribution of this family across many species suggests that these proteins have a basic function. In mammals, the TLP genes play important roles in the maintenance and functioning of neuronal cells, and mutating these genes can result in obesity, loss of vision and hearing, infertility, and insulin resistance (Coleman and Eicher, 1990; Heckenlively et al., 1995; Kleyn et al., 1996; Noben-Trauth et al., 1996). Tubby proteins may function as bipartite transcriptional regulators by binding to double-stranded DNA and activating transcription in the nucleus (Boggon et al., 1999; Santagata et al., 2001). Despite tubby domains being highly conserved, different animal TLP members are unable to compensate for one another in function (Ikeda et al., 1999).

Plant TLPs contain a conserved C-terminal tubby domain; however, unlike animal TLPs, most plant TLPs also contain highly conserved F-box domains in their N-terminus (Gagne et al., 2002; Lai et al., 2004). F-box containing proteins are involved in protein ubiquitination by acting as bridges between specific substrates and generic components of the SCF-type (Skp1-Cullin-F-box) or ECS-type (ElonginC-cullin-SOCS-box) E3 ubiquitin ligase complexes (Kile et al., 2002). Compared with the wide array of cellular functions identified for animal TLPs, the functions and mechanisms of plant TLPs are relatively unknown. In *Arabidopsis, At*TLP9 and *At*TLP3 are involved in ABA signaling during germination (Lai et al., 2004). *At*TLP9 also plays a role in responses to salt and drought stress (Bao et al., 2014). Overexpression of a TLP from chickpea was recently demonstrated to confer increased tolerance to salt, drought and oxidative stress (Wardhan et al., 2012). Furthermore, two studies in rice indicated that TLPs play important roles in host-pathogen interactions (Cai et al., 2008; Kou et al., 2009). Nevertheless, the highly conserved evolution of tubby (or tubby-like) proteins and their redundancy suggests that they play an indispensable role in plants.

As one of the most widely cultivated fruit trees, apple (*Malus domestica*) is also one of the most economically important woody plants (Hummer and Janick, 2009). The completion of the apple (*Malus domestica*) genome map offers the possibility of investigating the TLP gene family in this species (Velasco et al., 2010). A previous study showed that the expression of an apple tubby protein (TLP7) can enhance stress tolerance (Du et al., 2014). In this study, we performed a genome-wide search of TLP genes in the apple genome and analyzed their chromosomal distributions, functional domains, and expression patterns in different organs and processes. The three-dimensional structure of the tubby domain and conserved motifs were further modeled and characterized. Transcriptional profiling demonstrated organ-specific expression patterns for individual TLP genes. The expression of some *MdTLP*s were found to be sensitive to abiotic stress, which suggested that TLP family genes might be vital for the response and adaptation to abiotic stresses in apple. This study provides a foundation for the further functional analysis of plant TLPs.

## Materials and Methods

### Identification of *Md*TLPs in the Apple Genome

Two different approaches were used to identify *Md*TLPs in the apple genome. For the first approach, the published *Arabidopsis* TLP protein sequences were retrieved from the TIGR database^1^ and used as queries in BLASTP searches against the *Malus domestica* genome database (*Malus domestica* Genome v1.0^2^). Previous studies failed to detect *At*TLP4 in *Arabidopsis* using several experimental approaches, suggesting that it might be a pseudogene (Lai et al., 2004; Bao et al., 2014). Thus, we used 10 *Arabidopsis* TLP protein sequences as queries. To avoid excluding any possible candidates, the *E*-value cut-off was set to 0.001, as was done previously in a similar investigation (Li et al., 2011; Jia et al., 2013; Cui et al., 2015). Redundant sequences with the same accession numbers were removed from the data set. The retrieved sequences were then queried against the InterPro^3^ database to ensure the presence of tubby domains (Mitchell et al., 2015). For the second approach, the tubby-domain Hidden Markov Model Profile, which was downloaded from the Pfam^4^ database, was used as a query to search for all of the annotated proteins in the complete apple genome database^2^ using HMMER 3.0 (Eddy, 1998). The candidate proteins’ sequences were extracted by a Perl script and then examined for the tubby domain using the InterPro database. All of the sequences identified through these two methods were submitted to SMART^5^ to ensure the integrity of the tubby domain sequence. A BLASTN search (*E*-value ≤*E*^-100^) against the apple EST dataset was conducted to find the corresponding expression record for each putative family member^6^.

### Multiple Alignments and Phylogenetic Analysis

Multiple sequence alignments of the TLP amino acid sequences in apple (*Md*TLP1-9), *Arabidopsis* (*At*TLP1-3, *At*TLP5-11), rice (*Os*TLP1-14), and maize (*Zm*TLP1-15) were performed with ClustalX (Version 2.1^7^) (Saitou and Nei, 1987; Larkin et al., 2007). To make sure that all sequences were properly aligned, the sequences of the tubby domain were adjusted manually first, then let the other regions realigned, using software CLC Sequence Viewer 7^8^. An unrooted phylogenetic tree was constructed from the alignments of the full-length protein sequences according to the neighbor-joining method with 1,000 replications, and the phylogenetic tree was drawn with the MEGA5 program (Tamura et al., 2011).

### Chromosomal Location and Determination of the Exon/Intron Structure

Chromosomal location data were retrieved from apple genome annotations downloaded from the Genome Database for *Rosaceae*^9^. The chromosome map showing the physical location of all of the *Md*TLP genes was generated using a revised version of MapDraw (Liu and Meng, 2003). To explore the diverse exon–intron organizations of *MdTLP*s and *AtTLP*s (*AtTLP1*-*3, AtTLP5*-*11*), we compared the predicted coding sequences of *MdTLP*s and *AtTLP*s with their corresponding genomic sequences using GSDS software^10^ (Hu et al., 2015). The molecular weight and isoelectric point (pI) of the proteins were calculated with the ExPASY Compute pI/Mw Program^11^ (Wilkins et al., 1999).

### Identification of Additional Domains and Protein Subcellular Locations

To identify potential protein motifs and detect any additional domains outside the apple tubby domains, sequences of full-length *Md*TLPs were queried against the InterPro database^12^ (Mitchell et al., 2015). Protein subcellular locations were predicted using WoLF PSORT^13^, an extension of the PSORT II program (Horton et al., 2007).

### Motif Analysis and Homology Modeling of the Tubby Domain

All putative *Md*TLPs were analyzed by MEME (version 4.11.1^14^), a motif search tool for identifying conserved motifs of tubby domains (Bailey et al., 2009). To obtain the most significant conserved motifs in the nine *Md*TLPs, different numbers of motifs were tried with default parameters in normal mode. The identified motifs were annotated using SMART protein analysis software^15^.

The three-dimensional structure of the tubby domain was obtained by homology modeling using the website CPHmodels 3.2 Server^16^. Images were generated in the modeling package PyMOL v1.5^17^ (Nielsen et al., 2010).

### Searching for *cis*-Acting Elements in the Promoters of *MdTLP*s

To investigate *cis*-acting elements in the promoter sequences of *MdTLP*s, 1,500 bp of genomic DNA sequence upstream of the transcriptional start sites was obtained from the apple genome. The upstream sequences were subsequently scanned in the PlantCARE database^18^ for the presence of various *cis*-acting elements (Lescot et al., 2002).

### Sample Preparation and Total RNA Extraction

To investigate the expression of *MdTLP*s under abiotic stress, 3-year-old apple (*Malus sieversii*) seedlings were treated with either chilling at 4°C, 20% PEG6000, 100 mM H_2_O_2_, or 100 μM ABA for 0, 1, 3, 6, and 9 h. The leaves and roots from five individual plants were collected, placed into liquid nitrogen and stored at -80°C until further use. With respect to the samples used for organ-specific expression, different organs were collected and also stored at -80°C. Total RNA was isolated from the leaves using the CTAB procedure (Gasic et al., 2004). The RNA concentrations and A260/A280 ratios were determined using a NanoDrop Spectrophotometer (ND-1000 Spectrophotometer, Peqlab). The integrity of the RNA samples was examined with an Agilent 2100 Bioanalyzer (RNA Nano Chip, Agilent, Santa Clara, CA, USA). Suitable RNA was used for cDNA synthesis and qRT-PCR.

### Quantitative Real-Time PCR (qRT-PCR) Analysis

cDNA fragments were synthesized from total RNA using TransScript^TM^ One-step gDNA Removal and cDNA Synthesis SuperMix (TransGen Biotech, Beijing, China). To ensure the cDNA samples obtained were qualified, two stress-specific genes (DREB genes: *MDP0000147009* and *MDP0000218344*) in apple genome were selected as marker genes, which expressions have been demonstrated to be up-regulated under stress treatments (Zhao et al., 2012). Gene-specific primers for amplification from cDNA were designed based on target gene sequences using the Beacon Designers 8.10 software. The primer sequences used in this investigation are listed in **Supplementary Table S1**. qRT-PCR was performed with a CFX96 real-time system (Bio-Rad, USA) in a final volume of 20 μl containing 0.8 μl of cDNA, 10 μl of 2 × SYBR Premix Ex Taq (SYBR Green; Tiangen, China) and 0.8 μl of (10 μM) primers. The thermal cycling conditions were as follows: 44 cycles of 95°C denaturation for 15 s, 55°C annealing for 30 s and 72°C extension for 15 s. The apple *actin* gene was used as an internal control. The real-time PCR experiment was carried out at least three times under identical conditions. The relative expression levels were calculated as 2^-(Δ^*^C^*^toftreatment-Δ^*^C^*^tofcontrol)^. The relative expression levels of *MdTLP*s in stressed samples (1, 3, 6, and 9 h) were compared to the controls (0 h) with parametric one-way ANOVA at significance levels of *P* ≤ 0.05 and *P* ≤ 0.01.

## Results

### Genome-Wide Identification and Phylogenetic Analysis of *Md*TLPs in Apple

To identify the TLP protein-coding genes in apple, two approaches were used. For the first strategy, the peptide sequences of the TLPs of *Arabidopsis* were used as BLAST queries against the apple genome (*Malus domestica* Genome v1.0). To ensure that potential TLPs were not excluded and to obtain credible results, the *E*-value was set to 0.001, as done in a similar investigation (Li et al., 2011; Jia et al., 2013; Cui et al., 2015). Using this approach, 25 potential TLPs were identified in the apple genome. To determine whether these proteins contained tubby domains, the sequences were compared against the InterPro Database. Using this approach, eight potential TLPs were identified from the apple genome. For the second strategy, the tubby domain Hidden Markov Model Profile (Pfam01167) from the Pfam database^19^ was used to search the apple genome. A total of 10 putative TLPs were obtained. These sequences were also analyzed using the InterPro Database, and eight potential TLPs contained a tubby domain. All of the potential TLPs obtained by the above two strategies were submitted to the SMART database^20^ to verify the integrity of the tubby domain sequence and confirm the presence of apple TLP genes. Finally, eight genes were identified.

**FIGURE 1:**
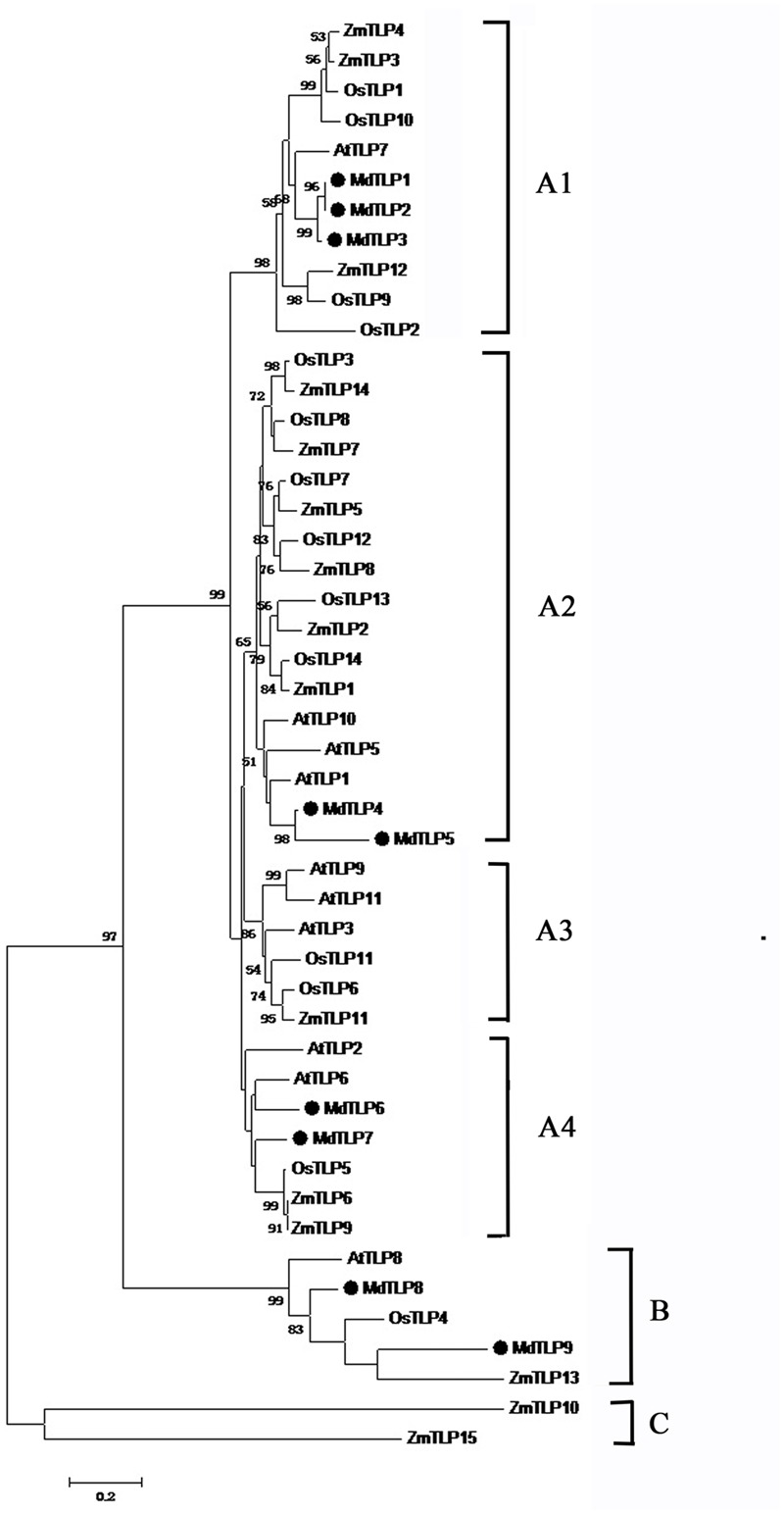
**Phylogenetic tree of putative *Md*TLPs and the TLPs from *Arabidopsis*, rice and maize constructed by ClustalX (Version 2.1).** Branch lengths indicate distance. The bootstrap support value of lower than 50% was hidden.

In our previous study, we cloned a TLP gene (named *MdTLP7*) from apple, whose expression increased significantly under cold stress (Du et al., 2014). However, the *Md*TLP7 gene sequence could not be identified in the published apple genome. To determine whether *MdTLP7* was present in the apple genome and to check the expression of this and other identified apple TLP genes, the gene sequences were searched against the apple EST database at NCBI^21^. In BLASTN analysis, each of the nine gene sequences hit several apple ESTs sequences (**Supplementary Table S2**), which indicated that these nine genes were genuinely expressed in apple. Therefore, we believe that the apple genome contains nine *Md*TLP genes (**Table 1**). To remain consistent with our previous studies, the *Md*TLP7 gene name was retained, and the other eight *Md*TLP genes were named *Md*TLP1-6 and *Md*TLP8-9 based on their distribution in the phylogenetic tree (**Figure 1**). The *Md*TLP peptides in length ranged from 269 to 693 amino acids, with predicted molecular weights between 29.9 and 78 kDa (**Table 1**).

**Table 1:** TLP family information for apple.

Group	Name	Gene identifier	ORF (bp)	Protein (aa)	theoretical Mw(kDa)/pI	Subcellular localization
**A1**	*Md*TLP1	MDP0000912429	810	269	29.9/9.43	Nuclear
	*Md*TLP2	MDP0000184528	1635	544	60.5/9.58	Chloroplast
	*Md*TLP3	MDP0000264408	1209	402	45.2/9.40	Chloroplast
**A2**	*Md*TLP4	MDP0000122158	1302	433	48.8/9.47	Nuclear
	*Md*TLP5	MDP0000303852	2082	693	78.0/9.44	Nuclear
**A4**	*Md*TLP6	MDP0000237033	1038	345	38.7/9.29	Nuclear
	*Md*TLP7	HM122708.1	1245	414	46.6/9.36	Nuclear
**B**	*Md*TLP8	MDP0000175577	1338	445	49.6/9.49	Nuclear
	*Md*TLP9	MDP0000320802	1431	475	52.8/9.53	Nuclear

*Gene identifiers with prefix MDP are from the Genome Database for Rosaceae (GDR, http://www.rosaceae.org/) and Gene identifiers with prefix HM from NCBI (National Center for Biotechnology Information). Protein subcellular locations were predicted using WoLF PSORT (http://psort.nibb.ac.jp/).*

Multiple sequence alignment showed that all plant TLPs had a highly conservative tubby domain at C-terminal almost with a conservative proline residue at the beginning (**Supplementary Figure S1**). To gain insight into the evolutionary relationships among all plant TLP proteins, a phylogenetic tree was constructed based on the full-length amino acid sequences from apple, *Arabidopsis*, rice, and maize (Lai et al., 2004; Yang et al., 2008; Yulong et al., 2016). All of the TLP encoding genes from the above four plant species were divided into three distinct groups: A, B, and C (**Figure 1**). Of 48 members, 41 plant TLPs belonged to group A and were further divided into the four subgroups A1–A4. Group B contained five members, and group C only contained two maize TLPs. Group A and group B contained TLP members from both dicot and monocot plants. The results showed that in some cases, the evolutionary relationship of plant TLPs between dicot and monocot was closer than that among dicot or monocot plants (**Figure 1**). For example, *Md*TLP9 was closer with *Zm*TLP13 than with *Md*TLP8 in group B (**Figure 1**). To check whether this result was valid, a detailed sequence alignment was checked (**Supplementary Figure S1**). Indeed, *Md*TLP9 had higher similarity with *Zm*TLP13 than *Md*TLP8 in sequences, especially in the segment of core α helix in tubby domain (indicated in the box of **Supplementary Figure S1**). These results suggested that the main characteristics of plant TLPs in group A and group B had been established before the dicot–monocot plants split. The TLPs from apple (*Md*TLPs) were distributed into two groups, group A, including A1, A2, and A4, and group B (**Figure 1**). The members in subgroup A3, *At*TLP3 and *At*TLP9 from *Arabidopsis*, have been studied in detail, and it has been indicated that they are involved in the stress response (Lai et al., 2004; Reitz et al., 2012, 2013; Bao et al., 2014). Whether *Md*TLPs in the different subgroups or different groups have similar functions requires further investigation.

### *MdTLP*s Chromosomal Location, Gene Structure, Additional Functional Domains, and Subcellular Localization Analysis

Based on the chromosomal distribution map of *Md*TLP genes generated in this study, all of the *Md*TLP genes were distributed across six of the seventeen apple chromosomes, including 8, 9, 10, 11, 15, and 17 (**Supplementary Figure S2**). Only chromosomes 8 and 11 contained two *MdTLP*s, while other *MdTLP*s were mapped to different chromosomes. *MdTLP1* and *MdTLP2* were both found on chromosomes 8, located near each other. They were also clustered together in the phylogenetic tree. To understand the possible structural evolution of *MdTLP*s, the intron-exon structure of *MdTLP*s and *AtTLP*s was analyzed in this study (**Supplementary Figure S3**). Intron/exon organizations for all *MdTLP*s were determined based on their exon position and gene length. The number of introns in the *MdTLP*s varied, ranging from 2 to 9 (**Supplementary Figure S3**). Unlike other *MdTLP*s, *MdTLP5* had much longer introns, although the number of introns (8) was similar to that of other *MdTLP*s. The *MdTLP*s in group B shared similar intron-exon structure distribution characteristics.

**FIGURE 2:**
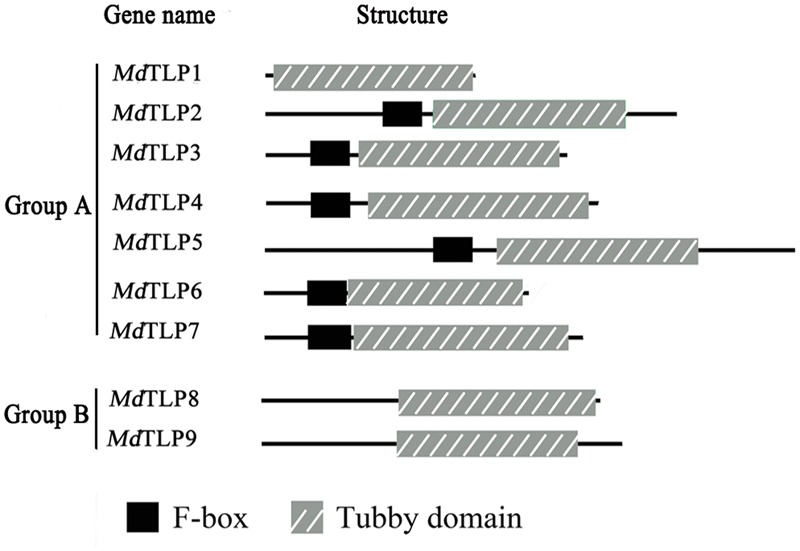
**Structure and organization of *Md*TLPs in apple.** The position of the signature tubby domain and F-box domain are shown. The gene name is indicated on the left.

Most *Md*TLPs in group A contained an additional functional domain, an F-box domain at the N-terminus (**Figure 2**). F-box-containing proteins constitute a large family in eukaryotes, which are characterized by a conserved F-box domain consisting of approximately 50 amino acids at their N-terminus. The appearance of both domains in one protein suggested that an interplay between tubby and F-box domains may play roles in physiological processes. Of nine *Md*TLPs, three members had no F-box domain (*Md*TLP1, *Md*TLP8, and *Md*TLP9) (**Figure 2**). Although these members were quite similar in patterns of protein structure, multiple alignments, and phylogenetic analysis showed that *Md*TLP1 was far from *Md*TLP8 and *Md*TLP9 (**Figure 1**; **Supplementary Figure S1**).

The subcellular location of *Md*TLPs was predicted, and the results are shown in **Table 1**. Most *Md*TLPs were predicted to be located in the nucleus. Only *Md*TLP2 and *Md*TLP3 in subgroup A1 were predicted to be located in chloroplasts. In mice, TLPs were mainly localized within the nucleus as transcription factors (Boggon et al., 1999). Here, we speculate that the nuclear-localized *Md*TLPs may also function as transcription factors. Future investigations are needed to experimentally confirm their location and transcription factor activity.

### Motif and Three-Dimensional Structure Analysis of the Tubby Domain

**FIGURE 3:**
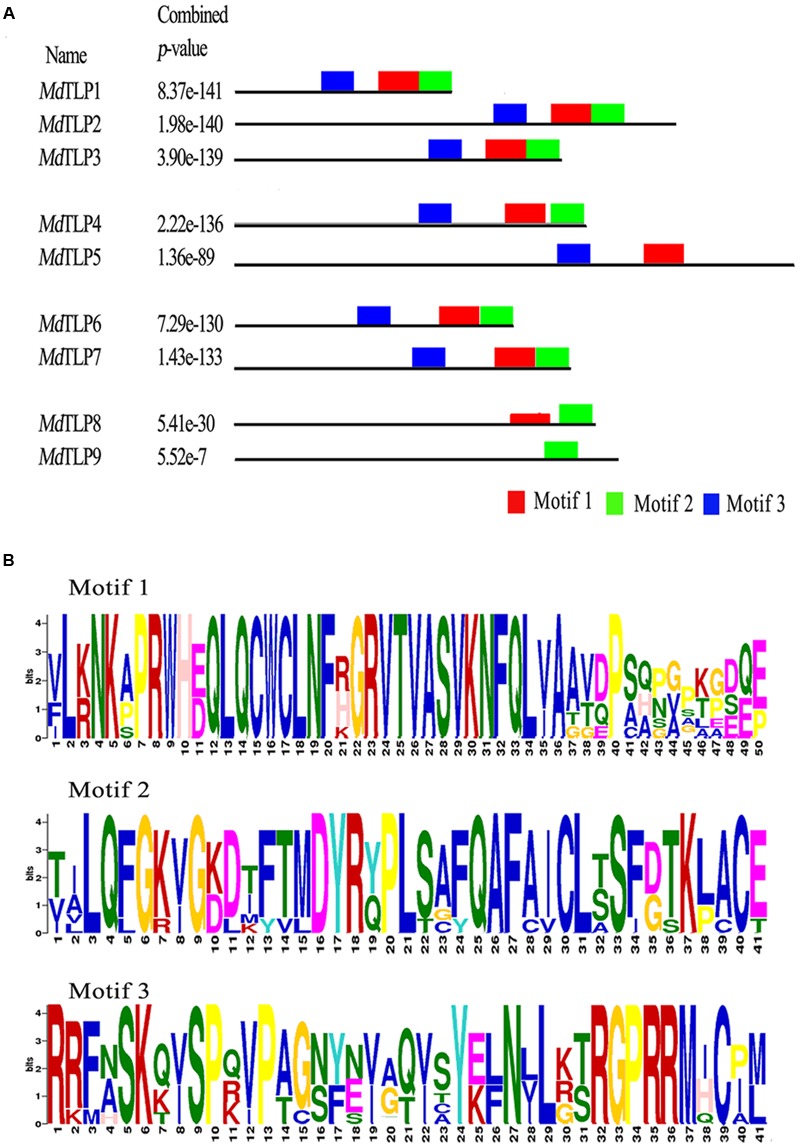
**Conserved motifs of nine predicted tubby domains of *Md*TLPs identified with the MEME program. (A)** Distribution of the identified motifs in the tubby domain of nine *MdTLP*s. **(B)** Consensus sequence for putative motifs.

All of the *Md*TLP peptide sequences were submitted to MEME^22^, and three types of motifs were identified in the tubby domain of *Md*TLPs (**Figure 3A**). The consensus sequences of motifs are shown in **Figure 3B**, with the height of each stacked letter representing the probability of that amino acid appearing at each position. Motif 1 and 2 were more highly conserved and usually located near the C-terminus of the polypeptide, harboring significant tubby domain characteristics similar to other plant and animal TLPs. Motif 3 often existed in the middle of the protein with a highly conserved amino acid sequence (RGPRRM), suggesting that this sequence may have an important biological function.

**FIGURE 4:**
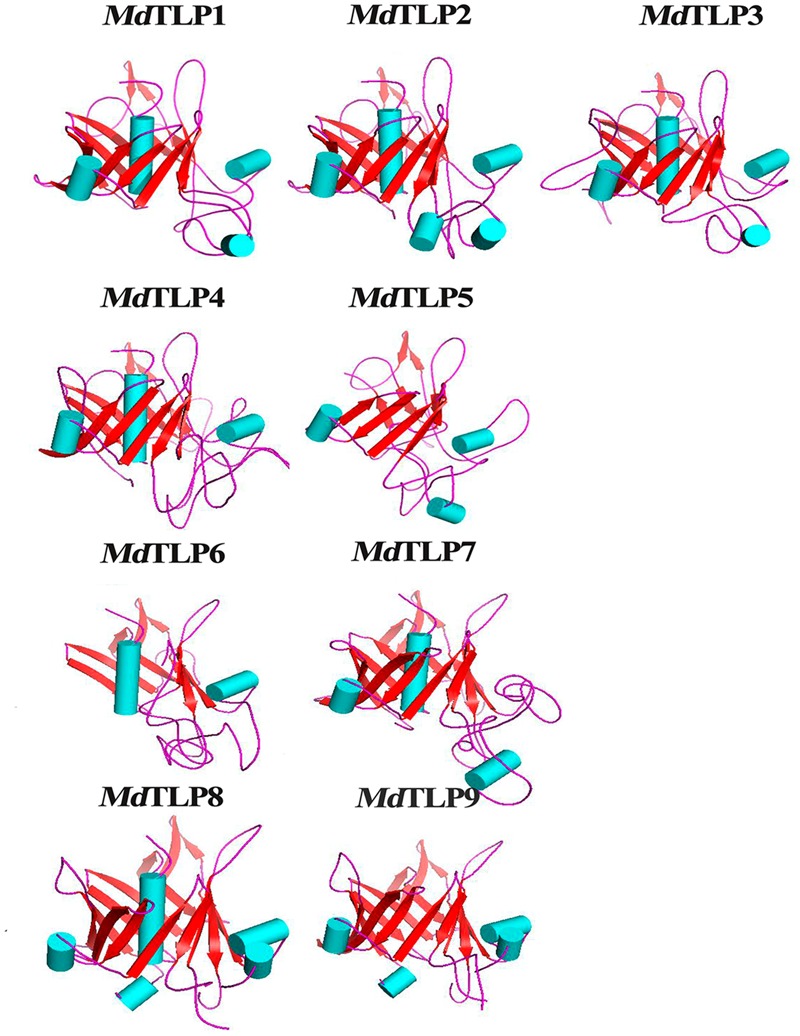
**Homology modeling of the tubby domain of *Md*TLPs.** The alpha helix is shown in light blue, and the beta fold is shown in red.

Three-dimensional structures of *Md*TLP tubby domains were established by homology modeling of a central α helix surrounded by a β barrel (**Figure 4**). Some *Md*TLP members had a typical tubby architecture with a closed β barrel formed by 12 anti-parallel strands and a central α helix, for example, *Md*TLP1, *Md*TLP2, *Md*TLP7, and *Md*TLP8. Other members contained an incomplete β barrel (less than 12 anti-parallel strands) and a central α helix, for example, *Md*TLP3, *Md*TLP4, and *Md*TLP6, which had 10, 11, and 6 anti-parallel sheets, respectively. *Md*TLP9 consisted of a complete β barrel without a central α helix, while *Md*TLP5 had an incomplete β barrel without a central α helix. Differences in the three-dimensional structures may lead to the functional diversification of different *Md*TLPs.

### Analysis of Promoter Sequences of *MdTLP*s

Transcriptional control of stress-responsive genes is a crucial part of the plant response to a range of abiotic and biotic stresses. Transcription factors have the potential to activate or repress genes through *cis*-acting sequences in promoter regions that respond to specific stresses (Singh et al., 2002). In plants, some TLPs play a role in responses to abiotic stress (Wardhan et al., 2012; Bao et al., 2014; Du et al., 2014). A search for putative *cis*-acting elements within the 1,500 bp of the genomic sequence upstream of the *MdTLP* 5′-UTRs was performed. Many stress-response related *cis*-acting elements were found in the promoter regions of *MdTLP*s, including ABREs, DRE, LTRE and MYB and MYC transcription factor elements (**Table 2**). MYB elements exist in all of the *MdTLP* promoters. MYB has been demonstrated to be involved in stress-induced drought, low temperature, salt, and ABA responses (Xiong et al., 2002; Dai et al., 2007). ABRE responds to drought and ABA via ABRE binding proteins in *Arabidopsis* (Hobo et al., 1999; Li et al., 2012). DRE binding proteins participate in drought, salt, low temperature, and ABA responses in rice (Zhang et al., 2009). LTRE primarily contributes to the regulation of low temperature responses in poplar (Jiang et al., 1996; Maestrini et al., 2009). The presence of abiotic stress-responsive elements suggests that *MdTLP*s may be regulated by various stresses. In addition to the *cis*-acting elements mentioned above, other types of *cis*-acting elements were also detected and are listed in **Supplementary Table S3**. Two promoters of identified *MdTLP*s were not analyzed because their promoter sequences could not be found in the apple genome database.

**Table 2:** Distribution of ABRE, DRE, LTRE, MYB, and MYC *cis*-acting elements in *MdTLP*s promoters.

Group	Gene	ABRE	DRE	LTRE	MYB	MYC	Total
A	*MdTLP1*	0	0	0	2	0	2
	*MdTLP2*	3	0	0	21	19	43
	*MdTLP3*	5	0	1	17	12	35
	*MdTLP4*	3	0	2	13	13	31
	*MdTLP5*	2	1	3	17	10	33
B	*MdTLP8*	1	3	3	24	22	53
	*MdTLP9*	3	2	2	22	20	49

### Expression of *MdTLP*s under Abiotic Stress, ABA, and H_2_O_2_ Treatments

**FIGURE 5:**
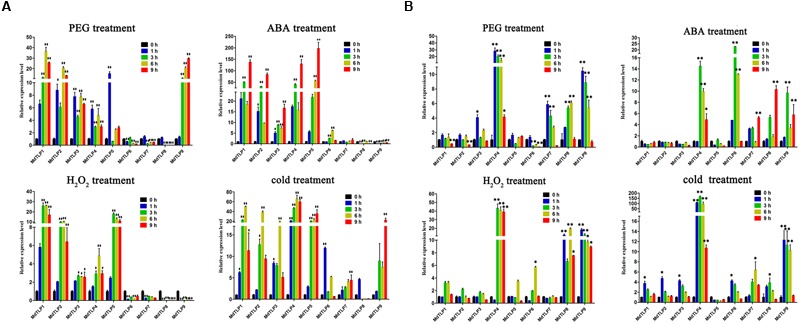
**Quantitative real-time PCR (qRT-PCR) for apple under PEG, H_2_O_2_, ABA or cold stress and exposure to 20 % PEG6000, 100 mM H_2_O_2_, and 100 μM ABA at 4°C for 0, 1, 3, 6, and 9 h.** Data were normalized to the expression level of *actin* gene. The mean expression value was calculated from three independent replicates. Vertical bars indicate the standard error of the mean. ^∗∗^*P* ≤ 0.01 and ^∗^*P* ≤ 0.05 compared with 0 h. **(A)** The expression levels of nine *MdTLP*s in apple leaves under different treatments. **(B)** The expression levels of nine *MdTLP*s in apple roots under different treatments.

Based on the promoter analysis results, *MdTLP*s may be associated with the abiotic stress response. Thus, to further investigate the potential functions of *MdTLP*s under abiotic stress conditions, cDNA samples were obtained using apple seedlings exposed to either PEG, H_2_O_2_, exogenous ABA, or cold stress for 0, 1, 3, 6, and 9 h. To detect the quality of cDNA samples, two stress-sensitive genes (DREB genes: *MDP0000147009* and *MDP0000218344*) were selected as marker genes. Similar with the previous report (Zhao et al., 2012), their expressions were significantly up-regulated under different stresses both in leaves and roots (**Supplementary Figure S4**). As to the expression of *MdTLP*s, almost all of the *MdTLP*s were also up-regulated in both leaves and roots under different stresses and exogenous ABA. During PEG treatment, six *MdTLP*s (*MdTLP1*-*5, MdTLP9*) were up-regulated significantly in leaves (**Figure 5A**). For example, the relative transcript levels of *MdTLP1* and *MdTLP9* were both up-regulated by approximately 35-fold compared with the control condition. Only three *MdTLP*s (*MdTLP6, MdTLP7*, and *MdTLP8*) were down-regulated under PEG stress. In the roots, five *MdTLP*s (*MdTLP3, MdTLP4*, and *MdTLP7*-*9*) were up-regulated significantly in response to PEG treatment (**Figure 5B**). Among them, the expression of *MdTLP4* changed maximum, reaching nearly 30-fold. Under cold stress, almost all *MdTLP*s in leaves and in roots showed significantly up-regulated transcript levels, suggesting that all *MdTLP*s are cold-responsive genes. Among these genes, *MdTLP4* expression increased nearly 70-fold in leaves in 6 h and about 150-fold in roots in 3 h. In response to H_2_O_2_ or ABA treatment, five members (*MdTLP1*-*5*) were significantly up-regulated in leaves, while in roots, the expression of *MdTLP4, MdTLP6, MdTLP8*, and *MdTLP9* showed a significant increase. Notably, *MdTLP4* was up-regulated in all the treatments both in leaves and roots.

### Expression of *MdTLP*s in Different Organs

**FIGURE 6:**
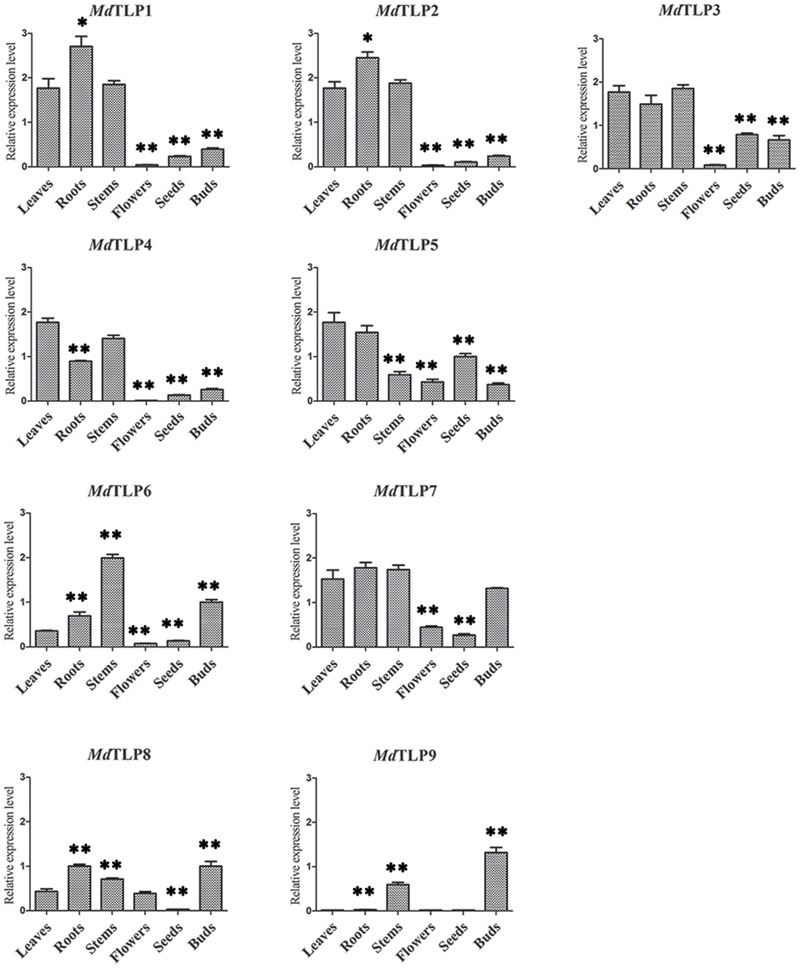
**Organ-specific expression pattern of *MdTLP*s.** The relative expression levels of *MdTLP*s in different organs were compared to the leaves with *T*-test at significance levels of ^∗∗^*P* ≤ 0.01 and ^∗^*P* ≤ 0.05.

To provide further clues to the putative roles of the *Md*TLP genes in apple development, the expression patterns of all 9 *Md*TLP genes were investigated by qRT-PCR in six different organs (leaves, roots, stems, flowers, seeds, and buds), and the results are indicated in **Figure 6**. Relatively high levels of *MdTLP*s were found in roots, stems, and leaves; in flowers, the transcript levels of *MdTLP*s were rather low. Compared with other *MdTLP*s, *MdTLP8*, and *MdTLP9* had low expression levels in most organs. *MdTLP1, MdTLP2, MdTLP3*, and *MdTLP4* were highly expressed in roots, stems, and leaves, with lower levels of expression in flowers, seeds and buds. Conversely, the *MdTLP6, MdTLP7, MdTLP8*, and *MdTLP9* transcript levels were relatively high in buds, which suggest that these genes play an important role in the sprouting process.

## Discussion

Tubby-like protein genes are members of a conserved gene family that has been identified in many species. Compared to animals, few TLPs have been functionally studied in plants. In the plant kingdom, TLPs have only been reported for a few species, such as *Arabidopsis* (Lai et al., 2004), rice (Liu, 2008), and maize (Yulong et al., 2016). The publication of the apple genome provided the opportunity to study the characteristics of this family in apple (Velasco et al., 2010). In this study, nine genes encoding TLP proteins were identified in apple, which is similar to the numbers reported in other plants, 11 for *Arabidopsis*, 14 for rice, and 15 for maize.

Phylogenetic analysis of TLPs from four plant species showed that all of the TLP genes were divided into three groups (**Figure 1**). The results in this study are similar to those of a previous report comparing the evolutionary relationship of *Arabidopsis* and rice TLPs (Yang et al., 2008). Here, *Md*TLPs were distributed into groups A and B. Group A included four subgroups, and group B contained five proteins: *Md*TLP8 and *Md*TLP9 in apple, *At*TLP8 in *Arabidopsis, Os*TLP4 in rice, and *Zm*TLP13 in maize. One apparent feature of all of the members of group B is that there is no F-box domain fused with the tubby domain at the N terminus. The members of this group may have originated from one gene in an ancestral species. Three motifs were identified in *Md*TLPs, and all three of these motifs could be found in all of the members of group A, except *Md*TLP5, which only possessed motifs 1 and 3. Motif 3 was not found in group B, *Md*TLP9 had only motif 2 and *Md*TLP8 had motifs 1 and 2. Motifs 1 and 2 were also found in the tubby domain of other species, such as *Arabidopsis* and rice (Lai et al., 2004; Kou et al., 2009). These two motifs were highly conserved among the TLPs from various organisms, suggesting that the two motif sequences were signatures of the tubby domain. However, motif 3 may be unique to apple.

Modeling of the three-dimensional structures suggested that the typical tubby domain of *Md*TLPs had a central α helix surrounded by a closed β barrel consisting of 12 anti-parallel sheets, as found in *Md*TLP1, *Md*TLP2, *Md*TLP7, and *Md*TLP8. In mammals, the crystal structure of the tubby domain was found to consist of a 12-stranded β barrel with a central hydrophobic α helix. The tubby domain binds readily to double-stranded DNA due to the β strands (5, 6, 7, 8, 9, and 10) of the β barrel (Boggon et al., 1999). A membrane-bound animal tubby protein translocates from the plasma membrane to the nucleus acting as a transcription factor to regulate the expression of genes when the cell receives a signal (Santagata et al., 2001; Carroll et al., 2004). However, some *Md*TLP members had an incomplete structure, such as an incomplete β barrel, either with or without the central α helix, which may contribute to different functions in apple.

Unlike animal TLPs, most plant TLPs have an additional F-box domain in the N-terminal protein sequence (Lai et al., 2004; Yang et al., 2008; Yulong et al., 2016) and are therefore also considered to be F-box proteins. F-box proteins have been reported to play roles in responding to abiotic stress (Stone and Callis, 2007; Yan et al., 2011; Maldonado-Calderon et al., 2012; Chen et al., 2014; Cui et al., 2015). The stress responses of plants are regulated by multiple signaling pathways, and there is a significant overlap between the patterns of gene expression that are induced by different stresses (Durrant et al., 2000; Schenk et al., 2000; Glazebrook, 2001; Knight and Knight, 2001; Seki et al., 2001; Chen et al., 2002). Gene induction by stress primarily occurs at the level of transcription and regulates the temporal and spatial expression patterns (Rushton and Somssich, 1998). Many transcription factors are involved in the expression of stress-related genes in plants. Often, several closely related transcription factors have the potential to activate or repress genes through *cis*-acting sequences that respond to specific stresses (Singh et al., 2002). In this study, the analysis of *MdTLP* promoter regions revealed a frequent occurrence of *cis*-acting elements, such as MYB/MYC, ABRE, DRE, and/or LTRE. These elements basically participate in drought, low temperature, and exogenous ABA responses (Xiong et al., 2002; He et al., 2012; Li et al., 2012).

To further study the response of *MdTLP*s to abiotic stress, qRT-PCR was used to determine the expression patterns of nine putative *MdTLP*s in apple. *Md*TLP genes were induced to varying degrees under different treatments, including drought, oxidative, exogenous ABA, and cold stresses (**Figure 5**). In particular, *MdTLP4, MdTLP6, MdTLP8*, and *MdTLP9* were up-regulated by more than 10-fold under exogenous ABA treatment at 3 h in roots. Four genes (*MdTLP1, MdTLP2, MdTLP4*, and *MdTLP5*) were up-regulated by more than 100-fold under exogenous ABA treatment at 9 h in leaves. Most *Md*TLP genes were also significantly up-regulated under drought, oxidative, and cold stresses in leaves and roots. Of *Md*TLPs, *Md*TLP1, *Md*TLP8, and *Md*TLP9 do not contain an F-box domain in the N-terminus. Therefore, we speculate that proteins containing an F-box domain or tubby domain may function in response to stress. When these two domains occur together, a synergistic effect may occur. In *Arabidopsis, AtTLP3* and *AtTLP9* were found to be up-regulated under abiotic stresses or ABA treatment (Lai et al., 2004), which two members belongs to subgroup A3 in phylogenetic tree. In this study, we found that the *Md*TLPs in subgroup A1, A2, A4, and group B were also sensitive to abiotic stresses. Especially, *MdTLP4* was significantly up-regulated under different stresses both in leaves and roots. Our result suggested that plant TLP members may participate in response to abiotic stress. Compared with the previous studies about plant TLP family, especially *Arabidopsis* TLPs, all the members of *Md*TLPs were comprehensive analyzed and confirmed to be sensitive to stresses in this investigation.

## Author Contributions

Designed the experiments: X-YW, S-SX, and J-NX. Performed the experiments: S-SX, J-NX, and Z-RZ. Analyzed the data: S-SX, J-NX, Z-RZ, and X-SC. Wrote the paper: X-YW, J-NX, and S-SX.

## Conflict of Interest Statement

The authors declare that the research was conducted in the absence of any commercial or financial relationships that could be construed as a potential conflict of interest.

## Footnotes

1 http://www.arabidopsis.org/

2 http://www.rosaceae.org/

3 http://www.ebi.ac.uk/interpro/

4 http://pfam.xfam.org/

5 http://smart.embl-heidelberg.de/

6 http://blast.ncbi.nlm.nih.gov/Blast.cgi

7 http://www.clustal.org/

8 http://www.clcbio.com

9 http://www.rosaceae.org/

10 http://gsds.cbi.pku.edu.cn

11 http://web.expasy.org/compute_pi/

12 http://www.ebi.ac.uk/interpro/

13 http://psort.nibb.ac.jp/

14 http://meme-suite.org/tools/meme

15 http://smart.embl-heidelberg.de/

16 http://www.cbs.dtu.dk/services/CPHmodels/

17 http://www.pymol.org/

18 http://bioinformatics.psb.ugent.be/webtools/plantcare/html/

19 http://pfam.xfam.org/

20 http://smart.embl-heidelberg.de/

21 http://www.ncbi.nlm.nih.gov/

22 http://meme-suite.org/tools/meme

## Supplementary Material

FIGURE S1 **Multiple alignments of the full-length proteins sequence of all plant TLPs.** The black asterisk over one amino acid indicates the start of the tubby domain. The locations of F-box domain is indicated with single solid lines in black above the sequences. The segment of core α helix in tubby domain was enclosed with a red rectangle.

FIGURE S2 **Locations of *MdTLP*s on apple chromosomes**.

FIGURE S3 **Intron patterns of *TLP*s from apple and *Arabidopsis***.

FIGURE S4 **Expression of the *DREB* genes (*MDP0000147009* and *MDP0000218344*) in leaves **(A)** and roots **(B)** under different treatments.** Data were normalized to the expression level of *actin* gene. Vertical bars indicate the standard error of the mean. ***P* ≤ 0.01 and **P* ≤ 0.05 compared with 0 h.

TABLE S1 **Sequences of the primers used in the quantitative real-time PCR analysis**.

TABLE S2 **The BLAST search result of each *MdTLP* against apple EST assemblies**.

TABLE S3 ***Cis*-acting element analysis of *MdTLP*s using the PLACE database**.

## Abbreviations

ABA: abscisic acid
ABRE: ABA-response element
DRE: dehydration-response element
DREB: dehydration responsive element binding protein
LTRE: low-temperature responsive element
qRT-PCR: Quantitative real-time PCR
TLP: tubby-like protein
