# Supplementary figures and images for: Genome-Wide Identification and Expression Analysis of the Tubby-Like Protein Family in the Malus domestica Genome
Source: Front Plant Sci. 2016 Nov 14;7:1693. doi: 10.3389/fpls.2016.01693 (PMC5107566; doi:10.3389/fpls.2016.01693)

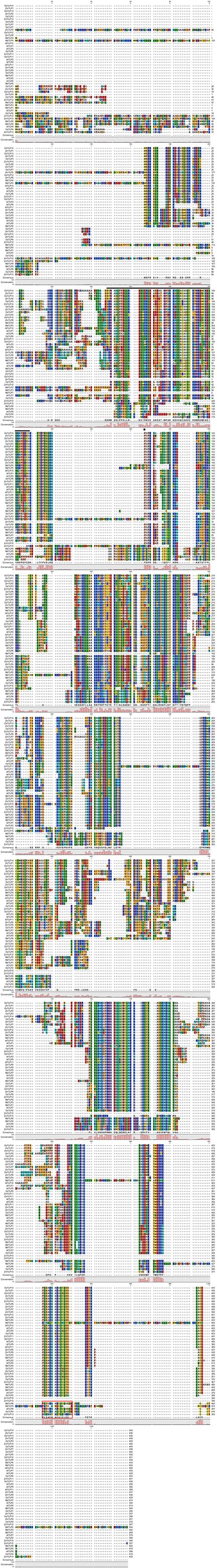

Supplement: FIGURE S1 — Multiple alignments of the full-length proteins sequence of all plant TLPs. The black asterisk over one amino acid indicates the start of the tubby domain. The locations of F-box domain is indicated with single solid lines in black above the sequences. The segment of core α helix in tubby domain was enclosed with a red rectangle. [file Image_1.TIF]

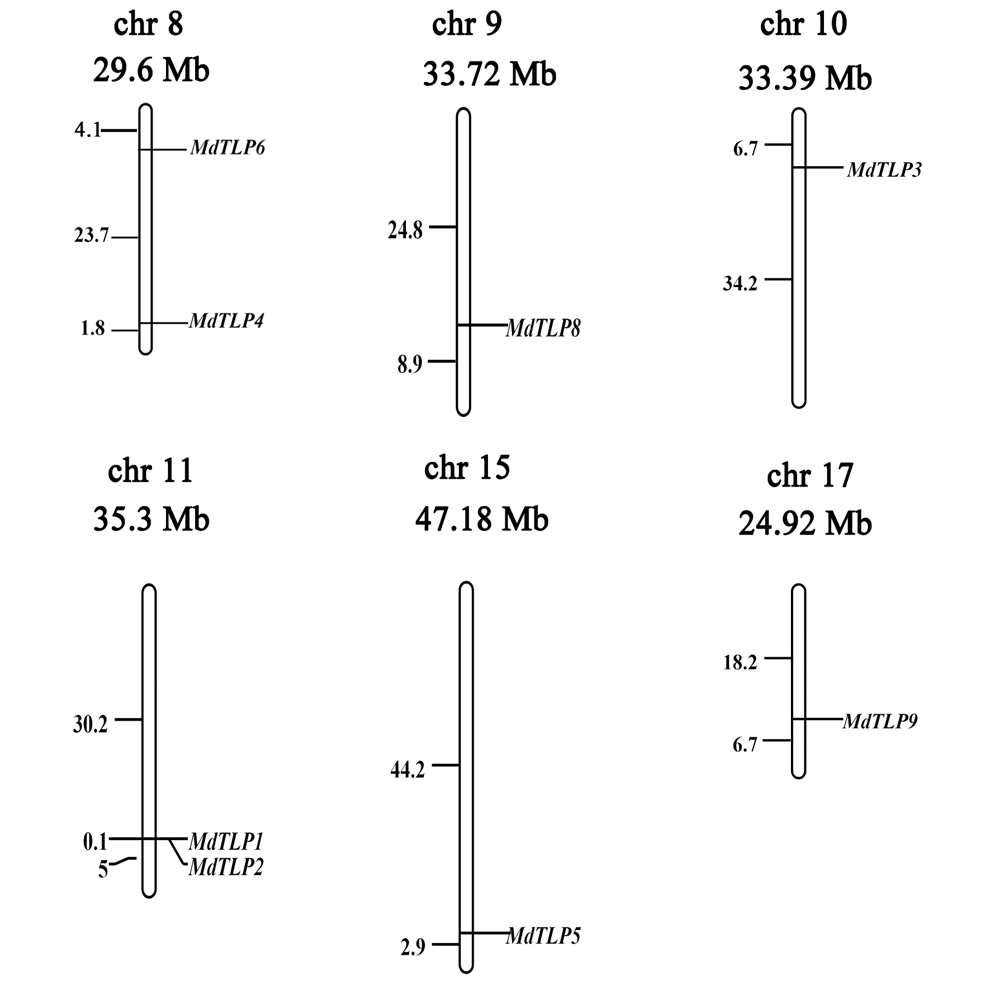

Supplement: FIGURE S2 — Locations of MdTLPs on apple chromosomes. [file Image_2.TIF]

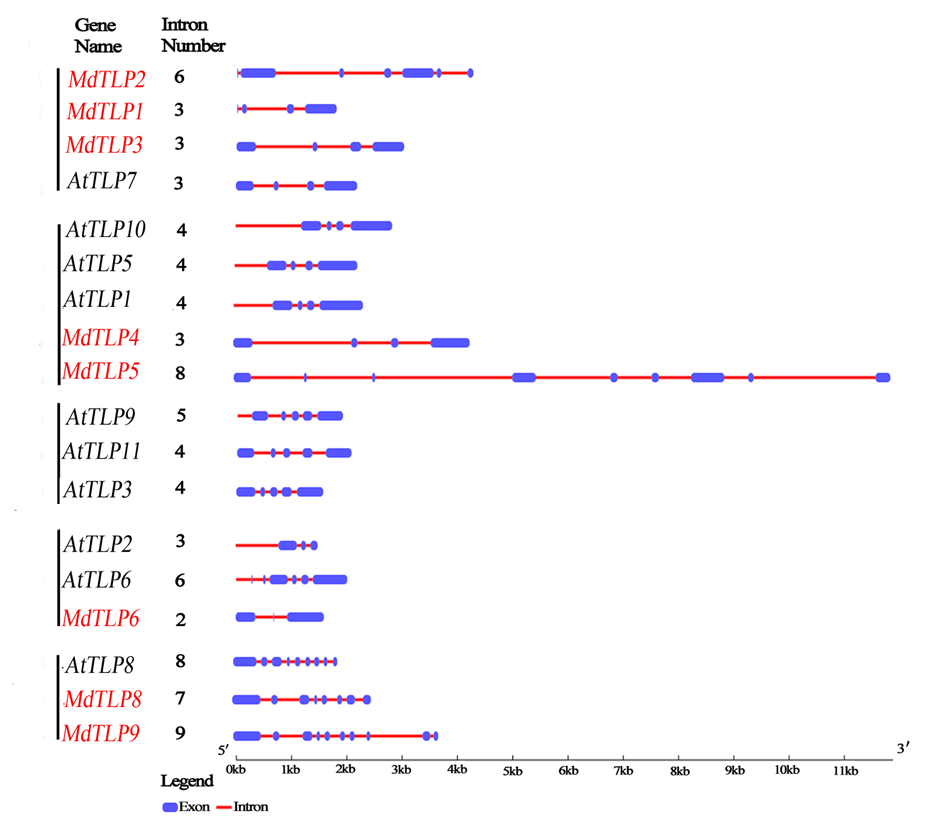

Supplement: FIGURE S3 — Intron patterns of TLPs from apple and Arabidopsis. [file Image_3.TIF]

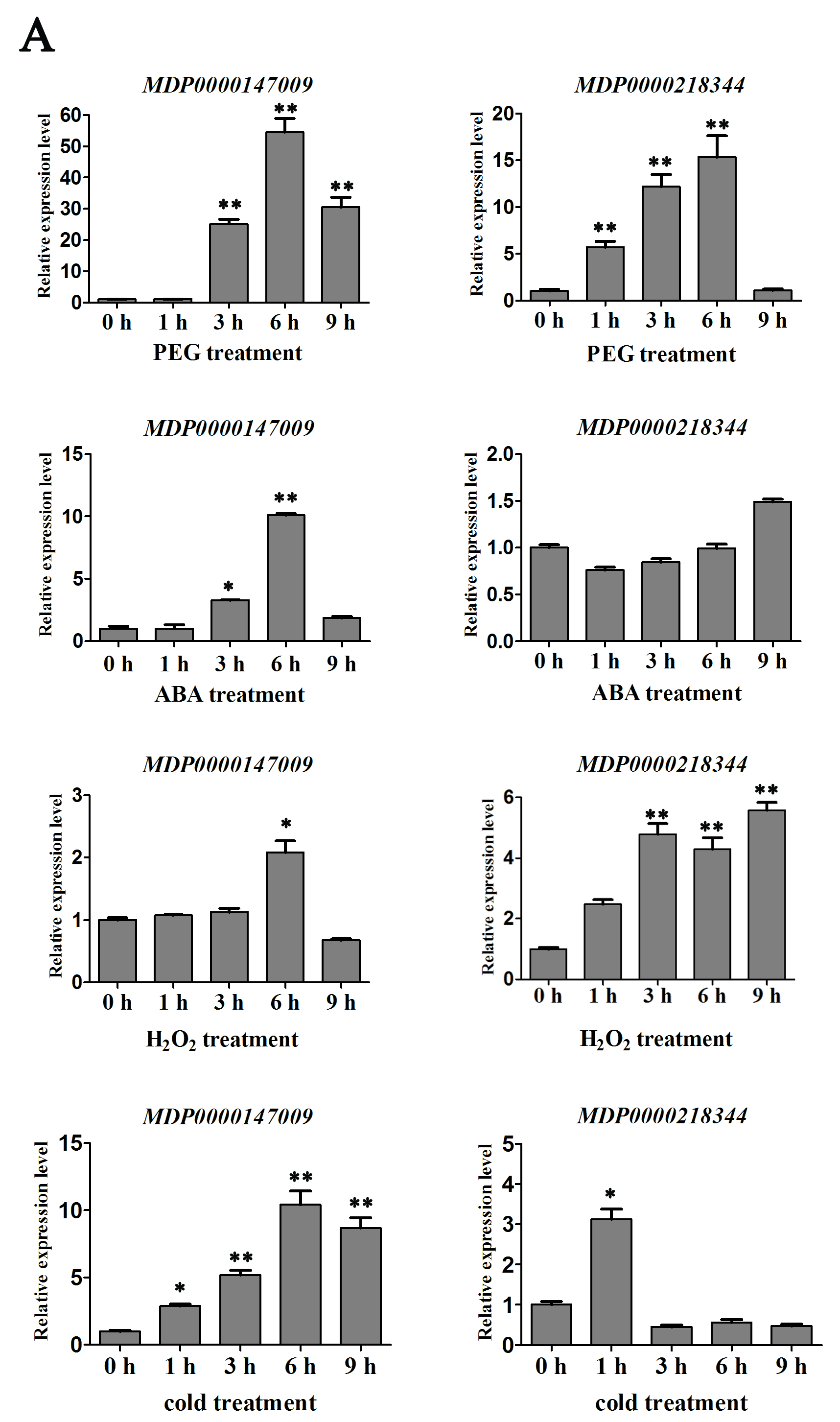

Supplement: FIGURE S4 — Expression of the DREB genes (MDP0000147009 and MDP0000218344) in leaves (A) and roots (B) under different treatments. Data were normalized to the expression level of actin gene. Vertical bars indicate the standard error of the mean. **P ≤ 0.01 and *P ≤ 0.05 compared with 0 h. [file Image_4.TIF]

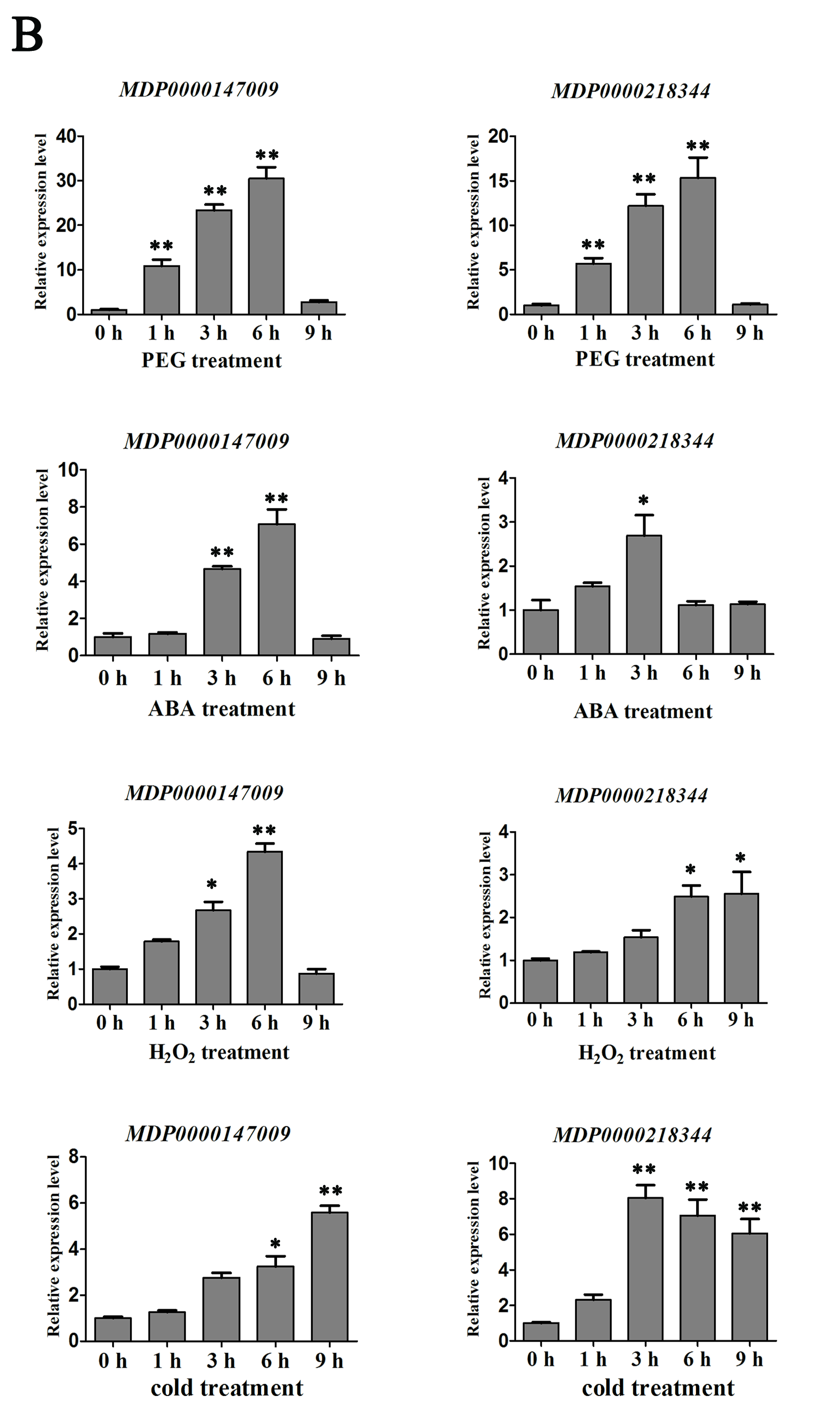

Supplement: Supplementary file 5 [file Image_5.TIF]
