# Supplementary material for: Genome-Wide Identification and Expression Analysis of the Tubby-Like Protein Family in the Malus domestica Genome
Source: Front Plant Sci. 2016 Nov 14;7:1693. doi: 10.3389/fpls.2016.01693 (PMC5107566; doi:10.3389/fpls.2016.01693)
Supplement: TABLE S1 — Sequences of the primers used in the quantitative real-time PCR analysis. [file Table_1.docx]

Table S1 Sequences of the primers used in the quantitative real-time PCR analysis

| Gene name | 3’-primer | 5’-primer |
| --- | --- | --- |
| *MdTLP1*  *MdTLP2*  *MdTLP3*  *MdTLP4*  *MdTLP5*  *MdTLP6*  *MdTLP7*  *MdTLP8*  *MdTLP9*  *MDP0000147009*  *MDP0000218344*  *Actin* | TCATCATTCACCGATAAG  CTCATCATTCACCGATAAGG  AGGTCTCTTACAAATTCAAC  CAGAATATGTTATCTCTATGG  TCATCTTCTCTGGTATGG  TATAGTGTTGCTACCGTATG  CTCCAATACAGTGCTTCAT  GCTATTCTCACGAGTTCC  GTCTACACTGCCTAACAA  GTAATCATACCGGCCAAGA  ATGGCAGAAGATTGGCTTAA  TGACCGAATGAGCAAGGAAATTACT | GGCTGTCATAGATTGTAA  GATATGTCATCAGCATCAAGT  TAGCCATAACTGGTAGAAG  ATTAAATCTACGGCTTGA  ATAAGTCTCGTGTCTCTT  CTTCTTGAACTGCTGACA  ATTTATCTTTCTCACCCTCAG  ATAGTAGTTACCTCCAGTGT  TGCTTCCTTCACTATCAC  AATAGTTGTAACCTCCATCTCC  CTGCTGGTTTGGATTGGTA  TACTCAGCTTTGGCAATCCACATC |
